# Tips for Effective Implementation of Virtual Reality Exposure Therapy in Phobias—A Systematic Review

**DOI:** 10.3389/fpsyt.2021.737351

**Published:** 2021-09-21

**Authors:** Marek Krzystanek, Stanisław Surma, Małgorzata Stokrocka, Monika Romańczyk, Jacek Przybyło, Natalia Krzystanek, Mariusz Borkowski

**Affiliations:** ^1^Clinic of Psychiatric Rehabilitation, Department of Psychiatry and Psychotherapy, Faculty of Medical Sciences, Medical University of Silesia in Katowice, Katowice, Poland; ^2^Department of Research and Development, Polfa Tarchomin, Warszawa, Poland; ^3^Multispecialistic Voivodship Medical Clinic in Katowice, Katowice, Poland; ^4^Abramowski 18th High School, Katowice, Poland

**Keywords:** agoraphobia, social phobia, specific phobias, exposure therapy, virtual exposure therapy, VRET, virtual reality, VR

## Abstract

**Background:** The high incidence of phobias and the limited accessibility of psychotherapy are the reasons for the search for alternative treatments that increase the availability of effective treatment. The use of virtual reality (VR) technology is an option with the potential to overcome the barriers in obtaining an effective treatment. VR exposure therapy (VRET) is based on a very similar rationale for *in vivo* exposure therapy. The study aimed to answer the question of how to perform exposure therapy in a virtual reality environment so that it is effective.

**Methods:** A systematic review of the literature, using PRISMA guidelines, was performed. After analysis of 362 records, 11 research papers on agoraphobia, 28 papers on social phobia and 10 about specific phobias were selected for this review.

**Results:** VRET in agoraphobia and social phobia is effective when performed from 8 to 12 sessions, on average once a week for at least 15 min. In turn, the treatment of specific phobias is effective even in the form of one longer session, lasting 45–180 min. Head mounted displays are an effective technology for VRET. Increasing the frequency of sessions and adding drug therapy may shorten the overall treatment duration. The effectiveness of VRET in phobias is greater without concomitant psychiatric comorbidity and on the condition of inducing and maintaining in the patient an experience of immersion in the VR environment. Long-term studies show a sustained effect of VRET in the treatment of phobias.

**Conclusion:** A large number of studies on in VR exposure therapy in phobias allows for the formulation of some recommendations on how to perform VRET, enabling the effective treatment. The review also indicates the directions of further VRET research in the treatment of phobias.

## Introduction

Phobic anxiety disorders are characterized by the occurrence of fear and anxiety in certain situations with little or no real threat, and a behavioral strategy to avoid those situations. Agoraphobia is an irrational fear of being out in the open space, in crowds, far from home, and of traveling alone. It is often accompanied or preceded by panic attacks. Social phobia, in turn, is an irrational fear of social situations and of avoiding them and specific phobias are fear and avoidance of specific objects or situations. All these phobias are common in the population. In the group of adults, the prevalence of specific phobias is estimated at 5–12% (1, 2), social phobia at 2.4% (3), and agoraphobia at 2.3% (4). All phobias may lead to a significant disability and impairment in everyday functioning, with the loss of social and professional roles (5).

Evidence from prospective studies suggests that anxiety disorders should be viewed as a chronic disorder that begins in childhood, adolescence or early adulthood, with a peak in middle age and a decline in old age (5). According to the 2015 Global Burden of Disease Study, anxiety disorders ranks ninth in the list of the largest contributors to global disability (6). In the case of social phobia, 37.6% of people diagnosed after 12 months found severe role impairment in at least one life domain, and an average number of 24.7 days out of role per 1 year was recorded (3). In the case of panic disorder with agoraphobia, 84.7% of people diagnosed after 12 months described severe impairment of the social role, and in the case of agoraphobia without a history of panic disorder, but with panic attacks, 39.0% reported severe impairment (7). These data show the urgent need to increase the availability of effective treatments.

The standard psychotherapy for agoraphobia and social phobia is cognitive-behavioral therapy (CBT) with the participation of a psychotherapist. Despite the convincing theoretical and empirical foundations, there appear to be barriers to the accessibility of this type of therapy in routine medical care. Neudeck and Einsle (8) mentioned structural barriers (e.g., time, insurance, or logistics) and barriers on the side of the therapist (e.g., negative attitude toward exposure therapy or insufficient knowledge of the method). These limitations hinder the accurate application of exposure techniques in clinical practice. These barriers pose a problem for patients, preventing them from receiving highly effective treatment (8). The use of virtual reality (VR) technology is an option with the potential to overcome these described difficulties. VR exposure therapy (VRET) is based on a very similar rationale for *in vivo* exposure therapy, however, in VR exposure, phobic stimuli are presented to the patient in a computer created artificial reality.

VR is a computer-generated reality that provides input to the user's sensory system and interacts with the user (9). Visual VR stimuli are presented through VR glasses [smartphone with 3D frames or a head-mounted display (HMD)] or by projection-based systems such as CAVE systems (automatic virtual environment in a cave), i.e., a room with up to six projection sides or Motek Caren system (10). The audio signal is input through speakers or head-phones, and optional tactile, or olfactory stimulation is possible but seldom provided. The goal of VR is to replace sensory stimuli from the real world and create an impression that a user is immersed in the real world experiencing. To interact with a user in real time, the VR system collects information about the user's position and head movements through sensors and input devices such as a head tracking system or a joystick.

To date, many clinical trials have been conducted, including randomized and controlled trials on the effectiveness of VRET in agoraphobia and social phobia. Due to a large amount of research, meta-analyzes assessing the above issue are also available in the literature. A summary of the most recent meta-analyzes on the use of VRET in the treatment of phobias is presented below.

In the meta-analysis by Wechsler et al. conducted in 2019 and involving 9 randomized and controlled clinical trials, the effectiveness of using VR in the treatment of agoraphobia and social phobia was assessed. It was shown that the use of VR in the treatment of social phobia compared to *in vivo* therapy did not bring any greater benefits (negative Hedges coefficient: −0.50). In the case of agoraphobia, no statistically significant advantage of *in vivo* therapy over VR was found (negative Hedges coefficient: −0.01). The authors indicated the need to conduct further randomized controlled clinical trials with the use of VR in order to expand the knowledge in this field (11).

Similar results were obtained by Carl et al. in a meta-analysis of 30 studies on the use of VR in the treatment of various phobias, including social anxiety and agoraphobia (12). These researchers showed a large effect size for VR compared to those who were not subjected to the intervention (positive Hedges coefficient: 0.90). In addition, an average to large effect size for VR was found compared to the psychological placebo conditions (positive Hedges coefficient: 0.78). The comparison of VR with conventional *in vivo* therapy did not show significant differences in the size of the effects (negative Hedges coefficient: −0.07). These results were relatively consistent across all analyzed disorders and they indicate that VR is effective and equal to conventional *in vivo* therapy as a medium for treating phobias (12).

In the most recent meta-analysis of 22 clinical trials with 703 participants by Horigome et al. the effectiveness of the use of VR in the treatment of social phobia was analyzed (13). The effectiveness of VR in treating social phobia was shown to be significant and sustained over a long observation period. Compared to *in vivo* exposure, the effectiveness of VR was similar after the intervention, but decreased in subsequent observation. The dropout rates of the participants showed no significant difference from the *in vivo* exposure results. Thus, the authors stated that VR is an acceptable method of treating patients with social phobia and has a significant long-term effect, although it is possible that its effectiveness will be reduced during long-term follow-up compared to conventional therapy.

Regarding the effectiveness of VRET in the treatment of specific phobias, a meta-analysis by Parsons and Rizzo (14) included 21 clinical trials involving 300 patients. VRET has been shown to be effective in reducing the symptoms of anxiety and phobias, especially in well-selected patients. The authors concluded that the use of VRET is effective in the treatment of anxiety and several specific types of phobias like social phobia, arachnophobia, acrophobia, agoraphobia, and aviophobia (14).

The impact of VRET on the behavior of patients with specific types of phobias in the real environment was also the subject of a meta-analysis of 14 studies conducted by Morina et al. (15). Behavioral evaluation results after treatment and during follow-up showed no significant differences between VRET and *in vivo* exposure (*g* = −0.09 and 0.53, respectively). The authors concluded that VRET cause significant changes in behavior in real-world situations (15). Also, in the last systematic review by Botella et al. (16), which included 11 randomized clinical trials, the effectiveness of using VRET in the treatment or support of the treatment of various types of phobias was assessed and found that applications using VRET have become an effective alternative that in terms of effectiveness can equal the results of traditional treatments for phobias (16).

The presented meta-analyzes confirm the effectiveness of VRET and its equivalence with *in vivo* exposure therapy. The authors decided to conduct their own review of studies to answer the question of how to perform exposure therapy in a virtual reality environment so that it is effective. The results of this literature review may provide clues for the planning of therapy protocols using VRET and for the construction of the VR environment for therapeutic means. They may also be considered to implement in subsequent projects of phobia exposure treatment using VRET.

## Materials and Methods

The review included clinical trials, as well as case series and case reports. The authors' assumption was that even in single case reports of patients treated with VRET, there may be data on VRET elements that affect the effectiveness of exposure therapy. PRISMA guidelines were used when preparing this systematic review (17). The criteria for including the study in the analysis were the presence of a diagnosis of agoraphobia, social phobia and specific phobias and VRET treatment. The analysis also included studies in which, apart from VRET, a different treatment method was used. Full-text publications available in English were included in the analysis. In each study, at least the baseline and endpoints of treatment efficacy had to be characterized.

The following medical databases were searched in the study: PubMed, Scopus, Web of Science, and Google Scholar (effective date 10/06/2021). The search was performed according to the PICO framework (P—patient, problem or population, I—intervention, C—comparison, control or comparator, O—outcomes). During our search, we used the following terms: “virtual reality” (Title/Abstract), “virtual exposure” (Title/Abstract), “agoraphobia” (Title/Abstract), “social phobia” (Title/Abstract), “social anxiety” (Title/Abstract); and “specific phobia” (Title/Abstract).

**Figure 1:**
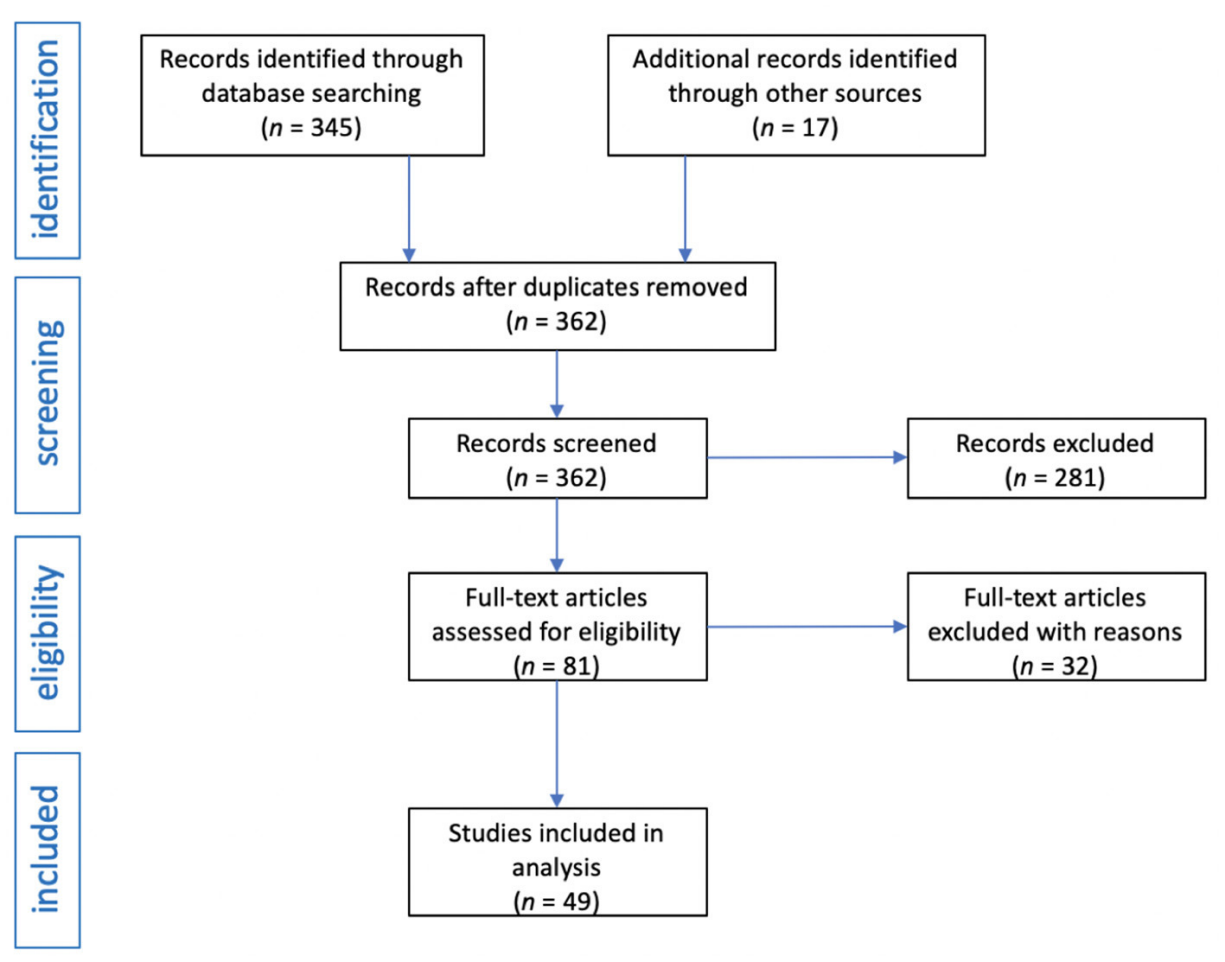
Flow diagram of studies analysis and selection for review.

The review was conducted independently by two investigators. After obtaining 345 records from the medical databases searched, the same terms were entered in the Google search engine and an additional 17 publications were obtained. When duplicate records were removed, 173 records were obtained for further analysis. In the next stage, an initial selection was carried out, excluding meta-analyzes, reviews, mini-reviews, systematic reviews, letters to the editor, editorials, comments, and errata. This pre-selection resulted in 81 publications. An in-depth selection was then performed and publications with only abstracts, papers in a language other than English, studies not directly related to the topic, and studies with methodological errors or data gaps were excluded. Ultimately, 49 clinical trials were included in the systematic review. The flow diagram of the analysis is presented in Figure 1.

To assess the risk of bias and study quality in quantitative studies, the Effective Public Health Practice Project's (EPHPP) Quality Assessment Tool for Quantitative Studies (QATQS) was used (18, 19). This tool enables quality evaluation of a wide range of study designs, including RCTs, observational studies with and without control groups and case studies. The instrument contains eight different sections, each with multiple questions: selection bias, study design, confounders, blinding, data collection methods, withdrawals and drop-outs, intervention integrity, and analyses. Each section receives a score of 1 (strong), 2 (moderate), or 3 (weak), and a final score is determined by the number of “weak” ratings. Strong rating is given to a study if there is no weak component score. Moderate rating is given with one weak component score. Weak rating is given with two or more component rating scores.

## Results

The tables below show the results of the systematic review of the literature on the use of VRET in the treatment of patients with agoraphobia (Table 1), social phobia (Table 2), and specific phobias (Table 3).

**Table 1:** Studies on the use of VR exposure therapy in agoraphobia.

**References**	***n***	**QATQS (points)**	**Characteristics of participants**	**Intervention used**	**Results**	**Conclusions**
North et al. (20)	60	2	Controlled clinical trial in patients with agoraphobia.	The subjects were assigned to the therapeutic group with the use of VR or to the control group without intervention.	Desensitization to phobic stimuli was demonstrated in the group using VR. The feeling of discomfort decreased with successive treatment sessions.	If side effects occur during VRET, they disappear in subsequent sessions and are not a reason for dropping out of therapy. Therapy with HMD.
Moore et al. (21)	9	3	Healthy subjects (8 women and 1 man); age: 22–27 years. Heart rate, skin conductivity, respiratory rate, and body temperature were analyzed before and after the VR intervention.	The display showed the environment: an elevator without people and crowded with people, a grocery store, a city square without people and with people, and a beach without people and with many people. The exposure to each environment lasted 2 min.	Most of the respondents experienced the realism of VR. There was an increase in skin conductance, an initial increase and then a decrease in heart rate, and insignificant changes in body temperature.	Even a 2-min VR exposure causes a feeling of realism accompanied by symptoms of neurovegetative excitation. Therapy with HMD.
Vinelli et al. (22)	12	2	Controlled clinical trial. Patients with panic attacks and agoraphobia.	Subjects were randomly divided into three groups: receiving CBT using VR (8 sessions) or conventional therapy who had experienced the traditional CBT (12 sessions) or control group without intervention.	Clinical improvement was achieved in the VR group during 8 and not 12 sessions as was the case with conventional therapy.	VRET is effective after 8 sessions. The duration of VRET may be shorter than *in vivo* therapy. VRET with HMD.
Alcaniz et al. (23)	1	3	Patient with agoraphobia.	Supporting psychological therapy of agoraphobia at home using VR on a personal computer (PC).	Not characterized. Authors conclusion was that he use of VR may be helpful in the treatment of agoraphobia.	VRET can be performed on a personal computer (PC).
Choi et al. (24)	40	2	Randomized controlled clinical trial. Patients with panic attacks and agoraphobia.	Subjects were randomized to either conventional CBT combined with VR (4 sessions) or a panic control program (12 sessions). The observation time was 6 months.	Significant improvement was demonstrated after treatment compared to the pre-treatment results in both treatment groups.	VRET may be effective in combination with CBT psychotherapy. No data available on the technique of VRET.
Botella et al. (25)	37	2	Controlled clinical trial. Patients with panic attacks with or without agoraphobia.	The subjects were qualified to the group using VR or the group with *in vivo* exposure or a control group. Clinical assessments were made before and after the treatment, and in the 12-month follow-up. The treatment programs consisted of 9 sessions weekly.	Clinical improvement has been demonstrated in both therapeutic groups.	VRET is effective when sessions are held over 9 weeks weekly. VRET on PC.
Pelissolo et al. (26)	92	2	Randomized controlled clinical trial. Patients with panic attacks and agoraphobia.	Subjects were randomized to either VR or classical CBT or the control group. The intervention consisted of 12 therapy sessions.	Clinical improvement was shown in both active treatment groups. There were no statistically significant differences in the effectiveness of the therapy between the two groups.	VRET is effective with 12 sessions of therapy. Therapy with HMD.
Malbos et al. (27)	18	2	Randomized controlled clinical trial. Patients with agoraphobia.	The subjects were classified into two groups: therapy with the use of VR or therapy with the use of VR and cognitive therapy. The subjects were exposed to 9 different virtual environments.	Questionnaires, behavioral tests, and physiological measurements indicated a positive influence of VR. The addition of cognitive therapy did not bring any significant additional benefits.	For VRET to be effective, it does not have to be combined with conventional CBT therapy. Therapy with HMD.
Meyerbroeker et al. (28)	55	3	Randomized controlled clinical trial. Patients with panic attacks and agoraphobia.	Subjects were randomly assigned to 4 sessions of CBT and then to 6 sessions using VR or 6 sessions with *in vivo* exposure, or a control group without intervention.	Both CBT and VR therapy were more effective than no intervention. In the panic disorder severity scale, *in vivo* exposure CBT was more effective than CBT with VR.	*In vivo* exposure may be more effective than in VR exposure in agoraphobia with panic attacks. Therapy with HMD or CAVE.
Castro et al. (29)	80	3	Clinical trial in patients with chronic agoraphobia.	Subjects were assigned to either VR therapy or conventional cognitive behavioral therapy or receiving treatment only. All subjects received anti-stress therapy. The observation period was 6 months.	All treatments were statistically effective after both treatment and 6 months of follow-up. The VR group showed clinical improvement in most of the variables measured during observation. The *in vivo* CBT group showed the highest dropout rates.	Patients treated with VRET are less likely to discontinue therapy than patients treated with CBT. Therapy with HMD.
Pitti et al. (30)	99	2	Randomized controlled clinical trial.	Subjects were randomly assigned to receive paroxetine and CBT, paroxetine and CBT and VRET, and paroxetine alone. Both combined groups received 11 CBT sessions, and one group also received 4 VR therapy sessions. Treatments were performed in individual sessions once a week for 3 months.	The three treatment groups showed statistically significant improvement. For some measures, the combined treatment groups showed greater improvement. The group exposed to VR showed greater improvement in the face of phobic stimuli.	VRET in combination with paroxetine is more effective than either of these methods alone. When combined with paroxetine, VRET is effective after 4 sessions a week. Therapy with HMD.

*The risk of bias and study quality assessed with the Effective Public Health Practice Project's Quality Assessment Tool for Quantitative Studies (QATQS) was presented as the global rating for each publication (1—strong, 2—moderate, 3—weak)*.

**Table 2:** Studies on the use of VR exposure therapy in social phobia.

**References**	***n***	**QATQS (points)**	**Characteristics of participants**	**Intervention used**	**Results**	**Conclusions**
Harris et al. (31)	14	2	Randomized controlled clinical trial. Students with social phobia.	Four weekly sessions using VR, each lasting 15 min.	It was shown that the applied intervention decreased the level of anxiety, feeling of discomfort, and the heart rate in the subjects.	VRET is effective with 4 sessions performed once a week. The session may be short, 15 min long. Therapy with HMD.
Roy et al. (32)	10	2	No information on randomization. Social phobia 6 women and 4 men Average age: 36.11 years Average duration of social anxiety disorder: 22.4 years.	12 sessions with the use of VR in the presence of a psychotherapist. Twenty minutes exposure to stressful situations during each session (identified during the first session). 6 subjects were included in the group cognitive-behavioral therapy and 4 in the VR therapy.	Improvement of the depressive symptoms; reduction of anxiety and avoidance of stressful situations in reality.	VRET is effective when exposed to a stressful situation for 20 min and after 12 sessions. Therapy with HMD.
Anderson et al. (33)	2	3	2 women with social phobia.	Each patient received a different VR intervention: weekly therapy (10 sessions) and intensive therapy (6 sessions) for 3 days.	Both subjects responded with a reduction in the perceived anxiety during public speaking.	VRET can be effective even after 6 sessions performed twice a day. It is also effective after a week with 10 sessions per week. Therapy with HMD.
Klinger et al. (34)	36	2	Controlled clinical trial. Patients with social phobia.	The subjects were assigned to the group of VR therapy or group CBT therapy. The virtual environments related to performance, intimacy, mindfulness and assertiveness. The intervention lasted 12 weeks and consisted of 12 sessions.	The results showed significant improvement in both treatment groups.	VRET is effective after 12 weekly sessions. VRET on PC
Grillon et al. (35)	10	2	Clinical trial with single-arm. Patients with social phobia.	The subjects were subjected to 11 sessions with the use of VR including situations containing phobic stimuli.	Reduction of social anxiety experienced during public speaking and reduction of the avoidance of eye contact with the audience were observed.	VRET is effective after 11 sessions. Therapy with HMD.
Anderson et al. (36)	11	3	Single-arm clinical trial. Patients with social phobia.	Individual sessions, including 4 sessions of psychoeducation and cognitive therapy and 4 sessions of exposure therapy using a virtual audience presented on a computer screen. A therapist was available in another room to answer questions and summarize up to 10 min after each session. Three months follow-up.	All self-report measures of social anxiety decreased; the improvement was maintained throughout the follow-up period. Participants reported that they were satisfied with the treatment, that they felt better after treatment, and that the computer program was user-friendly.	VRET is effective after 4 sessions. VRET on PC.
Wallach et al. (37)	112	2	Randomized controlled clinical trial. Patients with social phobia.	The subjects were randomly assigned to CBT in direct contact or with the use of VR or to the control group. The intervention included 8 sessions.	Reduction of anxiety in the group receiving active therapy was observed. During the study, twice as many respondents discontinued cognitive-behavioral therapy in direct contact than in VR group.	VRET is effective after 8 sessions. Therapy with HMD.
Donahue et al. (38)	20	1	Randomized controlled clinical trial. Patients with social phobia.	Subjects were exposed to a 4-min VR public speaking after receiving either quetiapine or placebo (double-blind) an hour earlier. A concurrent placebo/quetiapine VR exposition occurred 1 week later.	There was no significant effect of quetiapine on the outcome. However, quetiapine was associated with significantly increased heart rate and somnolence.	4 min VRET is less effective than drug therapy. Therapy with HMD.
Robillard et al. (39)	45	2	Randomized controlled clinical trial. Patients with social phobia.	Patients were assigned to either conventional CBT or VR therapy or to a control group. The intervention included 16 sessions.	The intervention groups showed similar significant reductions in social anxiety.	VRET is effective after 16 sessions. No information about the technic of VRET.
Lister et al. (40)	20	2	Randomized controlled clinical trial. Patients with phobia of public speaking.	The subjects were assigned to the active intervention group consisting of 4 VR sessions or to the control group.	Skin conductance and heart rate were shown to increase, suggesting that the virtual reality intervention was effective in triggering a fear response. VRET was found to reduce anxiety and negative beliefs about public speaking skills.	VRET is effective after 4 sessions. Therapy with HMD.
Wallach et al. (41)	20	2	Randomized controlled clinical trial. Patients with social phobia.	The subjects were assigned to the intervention therapy group using VR or cognitive therapy or CBT or waiting lists (WL). The intervention consisted of 12 sessions.	Cognitive therapy was no better than VR in cognitive measures, but was better than VR in one behavioral measure (LSAS fear). VR was more effective than cognitive therapy in terms of one behavioral parameter (reduction of fear in a behavioral task). There were no differences between the three treatments and all were superior to WL group.	VRET is effective after 12 sessions. Therapy with HMD.
Price et al. (42)	41	3	Randomized controlled clinical trial. Patients with social phobia.	The subjects were exposed to 8 sessions during which they were exposed to various social situations in VR: a conference room with 5–100 participants. Three factors characterizing the subject's immersion in virtual reality were analyzed: the sense of spatial presence, commitment, and the sense of reality.	Various components of the sense of reality were related to the experience of fear and the response to treatment with VR. Efficacy was significantly associated with the highest anxiety ratings reported by individuals during the exposure. The scale of involvement was the only factor that was significantly associated with response to treatment.	Eight sessions were required for VRET to be effective. The effectiveness of treatment is related to the sense of realism of the exhibition environment. Therapy with HMD.
Cornwell et al. (43)	32	2	A clinical trial involving 16 healthy people and 16 with social anxiety disorder.	The subjects were influenced by VR representing a conference room in which they were to deliver a short speech.	Patients with social phobia reported greater stress and anxiety than healthy people throughout the procedure.	The use of VR causes similar reactions to those accompanying reality, so that technology can be used in the treatment of social phobia. Therapy with 3dVisor.
Heuett and Heuett (44)	120	3	Controlled clinical trial. Students with phobia of public speaking.	The subjects were qualified for exposure to public speaking in virtual reality or during video visualization, or for a control group.	In both groups of active intervention, the feeling of fear of public speaking was reduced. Students who were exposed to VR reduced their fear of public speaking more than students exposed to video visualization.	It seems that the use of VR in treating social phobia is more effective than video visualization. Therapy with HMD.
Safir et al. (45)	49	2	Randomized controlled clinical trial. Patients with social anxiety. A follow-up of the study by Wallach et al. (37).	Subjects were assigned to either a VR or conventional CBT intervention group or a control group. The intervention consisted of 12 sessions. The observation period was 1 year.	After 1 year of observation, it was shown that the reduction of anxiety during social appearances was still maintained in both intervention groups.	The effectiveness of VRET requires 12 sessions. Therapy has long-term success. No information about the technic of VRET.
Anderson et al. (46)	58	2	Randomized controlled clinical trial. Patients with social phobia.	The subjects were assigned to the actual exposure group or the virtual or pending exposure group. People from active therapy groups participated in 8 sessions. The observation period was 12 months.	Subjects receiving active therapy improved compared to the waiting group. There were no differences between the active treatments in any process or outcome measure at any time, nor were there differences in the achievement of partial or complete remission.	Eight sessions are required for VRET to be effective. VRET is effective in treating social anxiety and the improvement is sustained for 1 year. No information about the way VRET was performed.
Gebara et al. (47)	21	2	Single-arm clinical trial. Patients with social phobia.	The subjects were exposed to 12 sessions of 50 min each, during which they were exposed to VR. The observation period was 6 months.	Improvement of social anxiety was observed in all scales and instruments used, including the follow-up period 6 months after the end of treatment. The average number of sessions was seven as participants quickly got used to the process.	VRET is effective on average after 7 sessions. The effect persists after the end of treatment. Therapy with HMD.
Kampmann et al. (48)	60	3	Randomized controlled clinical trial. Patients with social phobia.	The subjects were assigned to the *in vivo* or virtual exposure group or to the control group. The intervention included 10 sessions.	Compared to the waiting list, in active treatment groups social anxiety, perceived stress and beliefs related to avoidant personality decreased and the duration of speech increased. Subjects in the *in vivo* exposure group but not the VR group improved in terms of fear of negative judgment, speech performance, overall anxiety, depression, and quality of life compared to those on the waiting list. During the observation period, all improvements were significant for the *in vivo* exposure group. In the case of VR, only the effect of perceived stress was significant.	VRET may be slightly more effective than *in vivo* exposure. Therapy with HMD.
Anderson et al. (49)	28	2	Randomized controlled clinical trial. Patients with social phobia. A follow-up of the study by Anderson et al. (46).	Subjects completed 8 therapy sessions using VR or group conventional therapy.	It was shown that the 54% of subjects no longer met the diagnostic criteria for social phobia and 68% subjects reported that their condition improved.	For VRET to be effective, 8 therapy sessions are required. The effect lasts after the end of the treatment. No information how VRET was executed.
Stupar-Rutenfrans et al. (50)	35	3	Single arm clinical trial. Students with fear of speaking in front of an audience.	All subjects were exposed over 4 weeks to 8 sessions with the use of virtual reality imitating a lecture hall: without people and with a small and large audience.	It was shown that the fear of speaking decreased significantly after VRET sessions, and the decrease was strongest in participants with initially high levels of this anxiety. Participants with moderate to severe baseline anxiety levels had different anxiety patterns over time.	The effectiveness of VRET requires 8 sessions, conducted twice a week. The therapy is more effective in people who have a greater severity of anxiety in their first VRET sessions. Therapy with HMD.
Bouchard et al. (51)	59	2	Randomized controlled clinical trial. Patients with social phobia.	Subjects were randomly assigned to VR exposure (*n* = 17), actual exposure (*n* = 22) or waiting list (*n* = 20). Subjects receiving active intervention participated in 14 weekly sessions.	Social anxiety reduction was found in the active therapy group. Conducting therapy with exposure to VR was more effective than real exposure. The beneficial effects lasted 6 months.	VRET is effective with 14 sessions performed once a week. Therapy with HMD.
Kim et al. (52)	52	2	Controlled clinical trial. Patients with social phobia (*n* = 22) and healthy patients (*n* = 30).	The subjects were assigned to a VR intervention group or a control group. The intervention included 8 self-study sessions and lasted 2 weeks.	It was shown that the use of VR was associated with a reduction in anxiety and social anxiety and with an increase in speech time during public speaking.	VRET is effective after 8 sessions. A mobile VR application may be the treatment option at home. Therapy with HMD.
Kovar (53)	10	3	A comparative clinical trial without randomization. Patients with social phobia.	The subjects were divided into groups receiving therapy using VR and a group receiving psychotherapy. The intervention included 8 sessions.	Improvement in health and a reduction in the feeling of social anxiety was demonstrated in both therapeutic groups, but it was more pronounced in the group using VR.	VRET is effective with 8 sessions of therapy. VRET may be more effective than *in vivo* exposure. Therapy with HMD.
Denizci Nazligul et al. (54)	14	2	Randomized controlled clinical trial. Patients with social phobia.	The subjects were divided into a group using VR and a control group with conventional CBT. The intervention lasted 4 weeks and included 4 sessions.	Virtual reality and psychotherapy have been shown to be similarly effective in reducing public speaking anxiety.	VRET is effective after 4 sessions once a week. Therapy with HMD.
Geraets et al. (55)	15	2	Clinical trial with one therapy arm. Patients with severe social phobia.	The subjects were exposed to 16 sessions of cognitive-behavioral therapy with the use of VR. The observation period was 6 months.	VRET reduced social anxiety and improved the quality of life of respondents. During the observation period, symptoms of depression decreased.	VRET is effective in therapy involving 16 sessions. The treatment effect is maintained after the end of treatment. Therapy with HMD.
Yuen et al. (56)	26	2	A comparative clinical trial without randomization. Patients with social phobia.	The subjects were assigned to the intervention group using videoconferencing plus ACT (acceptance and commitment therapy) or VR + ACT. The intervention included 6 sessions.	Both treatment groups demonstrated a reduction in anxiety during social exposure. The satisfaction of the respondents was also comparable between the groups.	VRET is effective after just 6 sessions. VRET on PC.
Kahlon et al. (57)	27	3	A clinical trial in adolescents with social anxiety disorder.	Participants met for one 90-min training session 1 week after completing online initial therapy questionnaires. The treatment protocol included seven tasks of varying difficulty, ranging from 1 to 2 min, each with little or no preparation time. Adolescents used VRET only during the actual exposure tasks to avoid getting used to the virtual environment. Follow-up was 3 months.	The mixed-effect linear model revealed a significant reduction in social anxiety symptoms (Cohen's *d* = 1.53) before treatment, and the improvement was maintained throughout the follow-up period. Physiological data revealed a slight increase in heart rate during exposure tasks. Based on feedback from adolescents, the feasibility of the intervention was increased during the study.	The effectiveness of VRET requires appropriately intensified stimuli so as not to get use to digital environment too quickly. Therapy with HMD.
Lindner et al. (58)	23	2	A clinical trial. Patients with social anxiety in a routine medical care setting.	The subjects were exposed to sessions in VR. Follow-up was 3 months.	There was a significant decrease in public speaking anxiety after the first 3-h session. Multilevel modeling of in-session process measurements confirmed reduction of disastrous expectations and stress and the increase of quality of performance. Adherence to the online transition program that followed *in vivo* exposure was relatively poor, but symptoms continued to decrease. No changes were observed during the 3-month follow-up period.	VRET may be an effective form of continuing therapy *in vivo*. Therapy with HMD.

*The risk of bias and study quality assessed with the Effective Public Health Practice Project's Quality Assessment Tool for Quantitative Studies (QATQS) was presented as the global rating for each publication (1—strong, 2—moderate, 3—weak)*.

**Table 3:** Studies on the use of VR exposure therapy in specific phobias.

**References**	***n***	**QATQS (points)**	**Characteristics of participants**	**Intervention used**	**Results**	**Conclusions**
Triscari et al. (59)	65	3	Randomized controlled clinical trial in patients with aerophobia.	The subjects were randomized into three groups: - CBT (*n* = 22) - CBT + combined with eye movement desensitization and reprocessing therapy (CBT-EMDR) (*n* = 22) - CBT + VRET - 10 sessions of 2 h - Sessions 1–3: psychoeducation, cognitive and behavioral techniques and relaxation techniques. Flying education - Sessions 4–6: specific to each treatment group - Sessions 7–10: visit to the faith of flight control, flight simulation, and airplane flight.	All groups showed a reduction in the fear of flying. The performance measurements maintained a significant effect after the 1-year follow-up period.	10 VRET sessions combined with exposition *in vivo*. No information about the used device.
Levy et al. (60)	6	3	Single arm intervention clinical trial including acrophobic patients.	Patients were exposed to six sessions (two sessions per week) of VR exposure therapy. The first three were remote sessions, while the last three were traditional sessions with a therapist. The anxiety level, heart rate, presence, technical difficulties, and therapeutic alliance were analyzed.	It was shown that anxiety, presence, and therapeutic alliance were comparable in both VRET sessions and traditional therapy with the therapist.	VRET is effective in acrophobia after 6 sessions. Therapy with HMD.
Botella et al. (61)	63	2	Randomized controlled clinical trial in patients with small animal phobia.	- Participants were randomized to the group: - Exposure to real cockroaches (*in vivo*) (*n* = 31) - Exposure to spiders in VR (*n* = 32). - Patients were assessed prior to the session, then received one 3 h session. A reassessment was performed after the session and after 3 and 6 months.	Participants using VRET significantly improved on all outcome measures after treatment and at follow-up visits. When the two treatment conditions were compared, there were some post-treatment differences favoring participants who received *in vivo* exposure. However, these differences disappeared with 3- and 6-month follow-up.	VRET is effective in patients with small animal phobia after one session of 1 h. The effect lasts after the end of the therapy Therapy with HMD.
Gujjar et al. (62)	10	2	A controlled clinical trial involving patients with dental phobia.	The subjects were assigned to the VRET group or the educational advice group. The effectiveness of VRET was assessed by comparing the reduction in dental anxiety scores (measured 16 times over the 14-week study period and after 6 months of follow-up).	It has been shown to reduce the symptoms of dental phobia assessed on the Dental Anxiety scale and the Dental Fear scale, and to reduce behavior avoidance in VRET. Of the nine people who completed treatment, six (four in the VRET group and two in the education group) no longer had dental phobia after 6 months of follow-up.	Dental phobia resolves after 14 VRET sessions. Therapy with HMD.
da Costa et al. (63)	13	3	Single arm intervention clinical trial in women with driving phobia.	The respondents were exposed to 8 sessions with a computer game containing car driving scenarios covering several road situations. Participants' sense of presence, subjective suffering, and physiological responses were assessed during the eight VRET exposures. Participants' clinical features, cognitive abilities, and quality of life were also analyzed.	After VRET, there was a reduction in the incidence of distorted thoughts and state anxiety scores, as well as a slight improvement in quality of life. The subjective results of the discomfort, heart rate variability, and sense of presence scores confirmed that there was a sense of presence in the VRE environment.	VRET is effective in dromophobia after 8 sessions. VRET on PC.
Gujjar et al. (64)	30	1	Single-blind, randomized controlled clinical trial in patients with dental phobia.	Patients were randomized to VRET or an information booklet. A single VRET session with five scenarios was used. The measures of anxiety were assessed before and after the intervention as well as 1 week after and 3 and 6 months after.	It was shown that only patients in the VRET group showed a significant reduction in dental anxiety.	VRET is effective after 1 session with an in VR exposure of 45 min in which 5 different phobic scenarios were performed. Therapy with HMD.
Miloff et al. (65)	100	2	Randomized clinical trial in patients with arachnophobia.	Patients were randomized to one session of standard *in vivo* therapy or VRET The subjects were assessed using the behavioral approach evaluation test, a scale self-assessment of fear of spiders, depression, and quality of life before and after treatment, as well as after 3 and 12 months.	Behavioral avoidance and reported fear were significantly reduced in both groups after treatment discontinuation, with VRET approaching the strong treatment benefit of standard *in vivo* therapy over time.	VRET is effective in arachnophobia after just one session, lasting 3 h. The effect lasts after the end of the therapy. Therapy with HMD.
Kaussner et al. (66)	14	2	Randomized clinical trial in patients with fear of driving a car.	The subjects were randomized to VRET or waiting. The intervention included a medical and psychotherapeutic examination, two preparatory sessions with a psychotherapist, five sessions with VRET and a behavior avoidance test (BAT) in real traffic, a closing session and two further telephone assessments after 6 and 12 weeks.	The treatment helped to overcome fear and avoid driving. In the final BAT, all patients mastered the driving tasks they had previously avoided, 71% showed adequate driving behavior as assessed by the driving instructor, and 93% could maintain treatment success until the second control phone call.	5 VRET sessions are effective in dromophobia. The effect lasts after the end of the treatment. VRET was performed with high-fidelity fixed base driving simulator. The visual system comprises five image channels that provide a view of 300° horizontally and 47° vertically as well as a four-channels sound system.
Jiang et al. (67)	43	2	Randomized controlled clinical trial in patients with blood-injection-injury phobia.	Patients were randomized to the group:- VRET - Waiting list. One treatment session was used and was followed for 3 months.	Medium to large differences in catastrophic cognitions (probability [*g* = 0.88] and cost [*g* = 0.66] scores) were shown in favor of VRET. There were medium to large differences in favor of VRET in the post-injection anxiety and trauma subscale (MBPI *g* = 0.64–1.14) 1 week after treatment and after 3 months of follow-up, and in the MBPI syncope subscale (*g* = 0.84) and injections subscale medical anxiety test (*g* = 0.63) during observation.	One 90-min VRET session is effective in treating blood-injection-injury phobia. Therapy with HMD.
Lindner et al. (58)	25	3	A single-arm clinical trial in patients with arachnophobia.	One VRET session was used. The self-assessment of spider anxiety and quality of life was assessed twice before, 1 week and 2 weeks after treatment, and at 6 months.	It was shown that the symptoms of arachnophobia decreased both after treatment and during the 6-month follow-up period.	One session lasting 3 h is effective in the treatment of arachnophobia. The effect lasts after the end of the treatment. Therapy with HMD.

*The risk of bias and study quality assessed with the Effective Public Health Practice Project's Quality Assessment Tool for Quantitative Studies (QATQS) was presented as the global rating for each publication (1—strong, 2—moderate, 3—weak)*.

## Discussion

Taking for granted the previously demonstrated effectiveness of VRET in the treatment of phobias, the current review focuses on parameters regarding the duration of therapy, session duration, session frequency, combining VRET with other types of therapy as well as technology used in exposure therapy. It was assumed that the conditions of using VRET in studies in which in VR exposure proved to be an effective form of phobia treatment determined its effectiveness. They should be considered as guidelines for the development of protocols and applications for running VRET.

With regard to the number of sessions and the duration of therapy, as shown by the analysis of the literature in agoraphobia, the number of sessions should be from 8 to 12. On the other hand, in social phobia, the number of sessions ensuring the effectiveness of VRET is more diverse. Its efficacy was demonstrated in therapies performed with one-time session (57), and the highest number of sessions performed with great success was 16 (39). Most often, however, the number of sessions giving the effectiveness of VRET in the treatment of social phobia was, similarly to the treatment of agoraphobia, from 8 to 12 sessions. In the treatment of specific phobias, short therapies, most often consisting of one VRET session, were preferred, although longer protocols, including up to 14 sessions, were also successfully used.

The duration of one VRET session varies greatly depending on the study. If a single session therapy is to be effective, the exposure must last at least 60 min (57). It seems that for VRET sessions to be effective, they must last at least 15–20 min (31, 32), especially if at least 4 are performed during the therapy (31). As mentioned, VRET in specific phobias is most often conducted in the form of a one-time session, however, these sessions must be longer. Based on the analysis, the VRET session in specific phobia should not be shorter than 45 min (64), but most often they last longer, even up to 3 h (58, 61, 65, 67).

The conducted literature analysis shows that in agoraphobia the effectiveness of therapy is ensured by performing an average of one in VR exposure per week. It is similar in VRET in social phobia, and it is most often performed once a week. Perhaps performing VRET more than once a week may shorten the overall duration of therapy. In one study, it lasted 3 days with two VRET sessions a day (33). The possibility of reducing the duration of VRET therapy by increasing the frequency of sessions, for example twice a day, is a promising direction for further research.

Regarding the technology used in VRET, the most common are head mounted displays. They were used in 71.4% of the analyzed studies. Literature review demonstrates greater effectiveness of HMD technology over 2D image viewing (44). Contemporary technology offers portable HMDs that enable convenient home therapy (52). Such a set can also be a smartphone with an application for VRET installed on it. It will certainly allow for increased availability in the future, and thus may popularize VRET in the treatment of phobias.

Regarding the combination of different treatment methods, although VRET is an effective method used in monotherapy (11–16), however, it may be much more effective when combined with pharmacotherapy (30). When VRET is used with pharmacotherapy, the number of sessions can be shortened [e.g., 4 sessions in agoraphobia; (30)]. There are still too few studies on the augmentation of pharmacological treatment with VRET to draw conclusions about the number of sessions, their frequency and duration of a single session. This is a topic that requires further research. In addition to pharmacotherapy, VRET can be combined with *in vivo* exposure therapy, either as a pre-phase to *in vivo* therapy or as a follow-up to it. Also, in this case, it is necessary to conduct research on the possibilities and indications for combining these two types of exposure treatment.

An important issue is compliance with the eligibility rules for the exposure treatment of phobias in VR. Improper qualification for treatment without excluding comorbidity reduces the effectiveness of VRET (14). The analyzed studies and previously conducted meta-analyzes indicate that VRET is an effective exposure therapy in the treatment of phobias, but, if in addition to phobia, a patient suffers from another mental disorder, the effectiveness of exposure therapy is lower (28). This indicates the importance of proper qualification for VRET and avoidance of psychiatric comorbidity in order to ensure its effectiveness.

**Figure 2:**
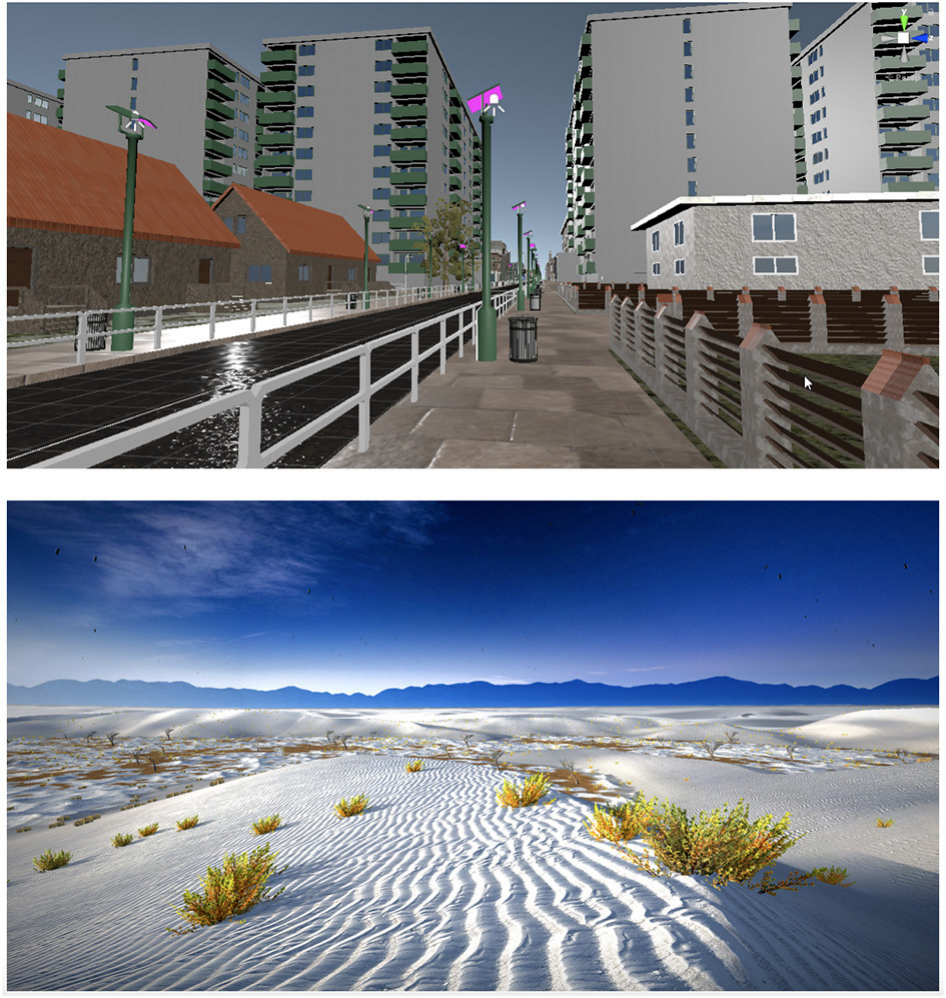
The virtual exposure environment must provide the patient with a sufficient level of realism for the VRET to be effective. The photos show examples of high-quality computer graphics of opened space exposure environment from the VR voice-BOT application, developed (photos made by MK). Before treatment, the patient determines the type of phobic environment, as well as customizes it depending on his preferences, specifying the time of day, weather and the type of exposure. Exposure in a three-dimensional graphics environment is enriched with three-dimensional sound, recorded in real conditions. Next, VR voice-BOT, thanks to the speech recognition system, conducts an exposure hierarchy with the patient, increasing the intensity of phobic stimuli in the environment previously defined by the patient and asking him to determine the subjective level of distress in the subjective units of distress scale (SUDS). During the exposure, the patient uses his own smartphone with the application installed on it, and a joystick, and moves freely in a virtual environment.

For the effectiveness of VRET, it is important for the patient to feel real and immersed in the environment provided by in VR exposure therapy (42, 43). With regard to the sense of immersion in virtual reality, it has been shown to occur very quickly. After just 2 min of using VRET, patients feel the realism of the virtual world (21). An important condition for the effectiveness of VRET is also the way of its conduct so that the patient does not get used to the digital VR environment too quickly without habituation to phobic stimuli. A way to counteract this familiarization with the digital environment may be the creation of many scenarios for the development of the exhibition environment (64). Also, the greater intensity of phobic stimuli may make it difficult to get used to the digital environment and to lose the sense of immersion in real experience (57). This is indirectly indicated by the greater effectiveness of in VR exposure in people with greater severity of phobias (50). In the technology of conducting therapy by automated voice-BOT therapeutic applications with a speech recognition system, it is possible to create an algorithm that increases the level of exposure to phobic stimuli depending on the speed of habituation to the VR exposure environment (Figure 2). The evidence that it is possible to provide a full sense of reality in digital reality at least at the level of *in vivo* exposure are the reports that in VR exposure is more effective than *in vivo* (48, 53). The more virtual reality will imitate reality in terms of graphic resolution, a variety of scenarios and their dynamic adaptation to the patient's behavior, the greater will be its effectiveness.

As indicated by the conducted analysis, VRET exposure may give a lasting effect. However, long-term efficacy has not been studied for more than a year it seems satisfactory. Safir et al. (45) showed that after 1 year of clinical improvement, the reduction of social phobia symptoms is still maintained, regardless of whether the therapy was performed with VRET or *in vivo* exposure (42). Similar in other studies that conducted long-term follow-up of patients after treatment, it was possible to demonstrate the durability of the treatment effect after the completion of VRET (46, 47, 49, 51). This may indicate no need for maintenance therapy with VR. To confirm that, in subsequent studies of the effectiveness of phobia therapy with VRET, long-term follow-up of patients after the completion of VR therapy should be considered.

## Conclusions

A large number of studies on in VR exposure therapy in phobias allows for the formulation of some recommendations on how to perform VRET, enabling the effective treatment. The conducted analysis of clinical trials allows to conclude that VRET in agoraphobia and social phobia is effective when performed from 8 to 12 sessions, on average once a week for at least 15 min. In turn, the treatment of specific phobias is effective even in the form of one longer session, lasting 45–180 min. Head mounted displays are an effective technology for VRET. Increasing the frequency of sessions and adding drug therapy may shorten the overall treatment duration. Moreover, the effectiveness of VRET in phobias is greater without psychiatric comorbidity and on the condition of generating and maintaining in the patient a sense of immersion in the VR environment.

Further studies should focus on the possibility of augmentation of pharmacological treatment with VRET, indications for combining in VR exposure with *in vivo* exposure, as well as the durability of VRET effects with possible maintenance therapy. In the future, it is also necessary to check the effectiveness of treatment protocols in which VRET is used more than once a week in terms of the possibility of reducing the total duration of treatment of phobias.

## Data Availability Statement

The original contributions presented in the study are included in the article/supplementary material, further inquiries can be directed to the corresponding author/s.

## Author Contributions

MK: conceptualization, data curation, and visualization. MK, SS, and MR: methodology. SS and MR: software. MK, MS, and MB: validation. SS, MR, MB, JP, MK, and NK: literature selection and analysis. MK, SS, MR, and JP: writing and original draft preparation. MK, MB, SS, MR, and NK: writing, review, and editing. MB and MS: supervision and funding acquisition. All authors have read and agreed to the published version of the manuscript.

## Funding

This study received funding from Polfa Tarchomin S.A. The funder was not involved in the study design, collection, analysis, interpretation of data, the writing of this article, or the decision to submit it for publication. 

## Conflict of Interest

The authors declare that the research was conducted in the absence of any commercial or financial relationships that could be construed as a potential conflict of interest.

## Publisher's Note

All claims expressed in this article are solely those of the authors and do not necessarily represent those of their affiliated organizations, or those of the publisher, the editors and the reviewers. Any product that may be evaluated in this article, or claim that may be made by its manufacturer, is not guaranteed or endorsed by the publisher.
